# Further benefits by early start of HIV treatment in low income countries: Survival estimates of early versus deferred antiretroviral therapy

**DOI:** 10.1186/1742-6405-7-3

**Published:** 2010-01-16

**Authors:** Kjell Arne Johansson, Bjarne Robberstad, Ole Frithjof Norheim

**Affiliations:** 1 University of Bergen, Department of Public Health and Centre for International Health, Research Group in Global Health: Ethics, Economics and Culture, PB 7804, 5020 Bergen, Norway

## Abstract

**Background:**

International HIV guidelines have recently shifted from a medium-late to an early-start treatment strategy. As a consequence, more people will be eligible to Highly Active Antiretroviral Therapy (HAART). We estimate mean life years gained using different treatment indications in low income countries.

**Methods:**

We carried out a systematic search to identify relevant studies on the treatment effect of HAART. Outcome from identified observational studies were combined in a pooled-analyses and we apply these data in a Markov life cycle model based on a hypothetical Tanzanian HIV population. Survival for three different HIV populations with and without any treatment is estimated. The number of patients included in our pooled-analysis is 35 047.

**Results:**

Providing HAART early when CD4 is 200-350 cells/μl is likely to be the best outcome strategy with an expected net benefit of 14.5 life years per patient. The model predicts diminishing treatment benefits for patients starting treatment when CD4 counts are lower. Patients starting treatment at CD4 50-199 and <50 cells/μl have expected net health benefits of 7.6 and 7.3 life years. Without treatment, HIV patients with CD4 counts 200-350; 50-199 and < 50 cells/μl can expect to live 4.8; 2.0 and 0.7 life years respectively.

**Conclusions:**

This study demonstrates that HIV patients live longer with early start strategies in low income countries. Since low income countries have many constraints to full coverage of HAART, this study provides input to a more transparent debate regarding where to draw explicit eligibility criteria during further scale up of HAART.

## Background

The optimal time to start treatment for HIV/AIDS has been a contentious issue since the introduction of Highly Active Antiretroviral Treatment (HAART). Initially a "hit hard and early" strategy was promoted [1]. Because of concerns about long term toxicity and fear of developing drug resistant viruses, delayed treatment starts were later recommended in clinical guidelines [2]. The delayed treatment policy implied that, in the absence of particular disease manifestations, treatment should not be started before CD4 counts dropped below 200 cells/μl. However, recent evidence indicates that this policy reduces survival compared to earlier treatment start. The World Health Organisation (WHO) revised the ART guidelines for resource constrained settings accordingly and re-introduced a "hit hard and early" strategy. In the revised 2009 guidelines, it is recommended that HAART is initiated on all HIV patients with CD4 counts below 350 cells/μl, regardless of symptoms [3]. Despite this change of recommendations, few low income countries have revised the national ART guidelines and many still recommend that initiation of HAART in asymptomatic HIV-infected persons are delayed until the CD4 count drops below 200 cells/μl [4]. Recent evidence from high income countries support even earlier initiation of treatment - before CD4 count drops below 350 cells/μl [5,6]. A clinical trial in Haiti recently demonstrated that deferring treatment until CD4+ T cell counts drops below 200 cells/μl, rather than providing HAART at CD4 counts between 200 and 350 cells/μl, increases death risk nearly four times [7]. However, there is little information to guide this important clinical decision in low income settings.

The debate regarding optimal timing of treatment start has tremendous implications for HAART demand, and subsequently, on the estimated treatment coverage in different settings. Towards the end of 2008, only 3 million people out of 33 million with HIV were given HAART [8]. In low income countries, treatment is still mainly provided to the sickest patients. Median baseline CD4 counts at initiation of HAART have been found to be between 100-150 cells/μl in several low income countries [9-15]. In contrast, a population based study from 2007 indicates that 42% of all HIV patients in Malawi had a CD4 cell count under 350 cells/μl, while 22% had under 200 cells/μl [16]. Shifts to an early treatment start strategy will increase the need for HAART, but few people actually receive HAART. Because of the huge gap between treatment coverage and needs, health outcomes from different treatment indications need to be assessed systematically.

Life years gained by different CD4 starting points is necessary information for making informed choices about early or late start of treatment. Studies in low income countries have found that patients starting HAART early (CD4 <350 cells/μl) have life expectancies from 9.4 to 17.2 life years and that life expectancies are 6.8 - 14.9 with late treatment strategies (CD4 < 200 cells/μl) [17-22]. Only one study adjust for lead time bias and report the size of the health benefit from the treatment; Cleary et.al. found that HAART yielded a net health benefit of 10 life years when it was initiated at the point when CD4 was below 200 cells/μl. The increase in the length of survival for patients starting treatment in earlier stages of HIV may reflect either earlier treatment initiation or delay in time of death. Therefore, to avoid overestimates of the actual impact of early treatment start, it is necessary to eliminate this potential confounding effect, which is often referred to as lead time bias. A recent eART-linc collaboration study found lead time to be as long as 4.6 life years [23]. Studies need to adjust for lead time bias and report net life years gained from different starting strategies in order to provide adequate information to make a rational clinical decision on this issue in a low income setting.

Our overall aim is to inform the ethical dilemma of choosing between providing HIV treatment to patients with potential best outcomes and giving fair chances to the more severely ill patients with less potential benefit. The objective of this study is therefore to estimate the life years gained with early and late treatment strategies in low income countries. In the absence of randomized clinical trials, we model life years gained by using best available evidence from observational studies.

## Methods

We did a structured literature review and combined findings in a pooled-analysis to find the best evidence underlying the different treatment strategies. Based on these findings, a Markov life cycle model was constructed to estimate the expected remaining life years with and without HAART stratified in three baseline CD4 strata at the time patients first present for care: CD4 <50 cells/μl or 50-199 cells/μl or 200-350 cells/μl. The model can be applied on any population, and we test it on a Tanzanian HIV population since information on HIV prevalence and life tables were easily available [24,25]. We eliminate lead time bias by calculating survival with and without treatment for three CD4 strata, representing three different stages of the disease, and subtracting a stratified net health benefit for each CD4 strata.

**Figure 1:**
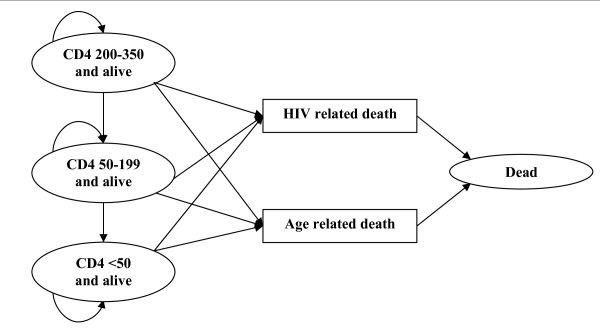
**Markov state transition diagram illustrating the life cycle model used to calculate the effects of the three alternative interventions**. Each oval represents a health state in the Markov model. During each successive year, patients may continue in their present health state, die from HIV or age related death (and transition to a death state). Patients not initiating HAART may also jump to lower CD4 states.

The set of mutually exclusive health states in the model and the various events that can occur in the history of HIV are illustrated in Figure 1. In our model, all patients have CD4 counts below 350 cells/μl and are assigned to the initial health states called "CD4 200-350 and alive", "CD4 50-199 and alive" or "CD4 <50 and alive". The Markov-cycle tree model is evaluated by expected value simulation, and a cycle length of one year is applied. At the end of each cycle, patients may continue in the initial health state or move from one state to either of the two events called "HIV related death" or "Age related death" or to a lower CD4 stratum, according to transition probabilities (see below). We considered 100 Markov cycles (years) to ensure that the whole cohort has moved to the death state by the end of the analysis. We incorporate dynamic changes in CD4 cell count occurring after patients enter the model for those not initiating HAART. We did not allow HIV patients initiating HAART to jump down to lower CD4 strata since studies indicate a large CD4 recovery the first four years after HAART initiation [26]. The effect from this CD4 recovery will be incorporated in the pooled HIV-related mortality-rates.

### Model parameters

We assume that patients presenting for care have a mean age of 35 years because this agreed with the characteristics of the underlying HIV demographics in the region and mean age of the studies included in the pooled-analysis (tables 1 and 2). We only included HIV populations with CD4 counts < 350 cells/μl.

**Table 1:** Pooled-analysis of one-year HIV-related mortality rates for HIV patients not taking HAART stratified in three baseline CD4 strata.

Study	n	Median age	Weight (%)	Absolute risk of death per year (95% CI)
**Baseline CD4 count <50**				

Thailand (40)	345	NA	1.00	0,796 (0,697 - 0,894)

**Weighted mean (weighted CI)**	345			**0,796 (0,697 - 0,894)**

				

**Baseline CD4 count 50-199**				

South Africa (39)	361	NA	0.22	0,192 (0,154 - 0,234)
Thailand (40)	77	NA	0.05	0,216 (0,135 - 0,298)
South Africa (41)	668	32	0.41	0,270 (0,230 - 0,330)
Thailand (42)	227	26-29	0.14	0,278 (0,250 - 0,313)
Uganda (46)	78	30	0.05	0,532 (0,421 - 0,643)
Gambia (45)	221	30	0.14	0,941 (0,801 - 1,080)

**Weighted mean (weighted CI)**	1632			**0,355 (0,335 0,375)**

				

**Baseline CD4 count 200-350**				

South Africa (39)	206	NA	0.14	0,088 (0,057 - 0,128)
Thailand (40)	192	NA	0.13	0,077 (0,047 - 0,107)
South Africa (41)	326	32	0.23	0,080 (0,050 - 0,120)
Thailand (42)	467	26-29	0.32	0,104 (0,100 - 0,128)
Uganda (46)	81	30	0.06	0,175 (0,092 - 0,258)
Gambia (47)	175	30	0.12	0,207 (0,160 - 0,254)

**Weighted mean (weighted CI)**	1447			**0,109 (0,093 - 0,125)**

NA = Not Available

**Table 2:** Pooled-analysis of one-year HIV-related mortality rates for HIV patients initiating HAART at CD4 counts <50, 50-199 and 200-350 cells/μl.

Study	n	Median age	Weight (%)	Absolute risk of death per year (95% CI)
**Baseline CD4 count <50**				

**Year 1 on HAART**				
ART LINC (12)	1474	36	0.18	0,062 (0,049 - 0,074)
Senegal (24)	96	37	0.01	0,179 (0,115 - 0,272)
South Africa (30)	155	31	0.02	0,182 (0,13 - 0,253)
Cambodia (ref)	416	33.6	0.05	0,130 (0,100 - 0,170)
Botswana 2 (Wester)	43	36	0.01	0,240 (0,116 - 0,355)
Côte d'Ivoire (23)	2655	36	0.33	0,230 (0,200 - 0,250)
Botswana 1 (Mujugira)	349	35	0.04	0,340 (0,288 - 0,388)
Zambia (29)	2797	35	0.35	0,308 (0,291 - 0,325)

**Weighted mean (weighted CI)**	7985			**0,224 (0,215 - 0,233)**

**Year 2+ on HAART**				
Senegal (24)	79	37	1.00	0,088 (0,026 - 0,150)

**Weighted mean (weighted CI)**	79			**0,088 (0,026 - 0,150)**

				

**Baseline CD4 count 50-199**				
**Year 1 on HAART**				
ART LINC (12)	2022	36	0.10	0,030 (0,022 - 0,037)
West-Africa (25)	232	34	0.01	0,050 (0,026 - 0,087)
Senegal (27)	176	38	0.01	0,074 (0,052 - 0,105)
Botswana 2 (Wester)	87	36	0.00	0,097 (0,035 - 0,156)
Haiti (28)	1004	NA	0.05	0,140 (0,117 - 0,162)
Senegal (24)	177	37	0.01	0,131 (0,089 - 0,190)
South Africa (30)	132	31	0.01	0,086 (0,049 - 0,151)
South Africa (39)	81	NA	0.00	0,069 (0,031 - 0,133)
Côte d'Ivoire (23)	7556	36	0.39	0,090 (0,070 - 0,110)
Zambia (29)	6787	35	0.35	0,140 (0,132 - 0,148)
Malawi (ref)	1298	34.9	0.07	0,190 (0,170 - 0,190)

**Weighted mean (weighted CI)**	19552			**0,110 (0,106 - 0,114)**

**Year 2+ on HAART**				
Malawi (ref)	1051	34.9	0.87	0,09 (0,070 - 0,110)
Senegal (24)	154	37	0.13	0,06 (0,025 - 0,095)

**Weighted mean (weighted CI)**	1205			**0,088 (0,072 - 0,104)**

				

**Baseline CD4 count 200-350**				
**Year 1 on HAART**				
ART LINC (12)	940	36	0.23	0,011 (0,004 - 0,017)
West-Africa (25)	408	34	0.10	0,017 (0,006 - 0,038)
Senegal (24)	122	37	0.03	0,058 (0,028 - 0,119)
South Africa (39)	110	NA	0.03	0,014 (0,002 - 0,049)
Zambia (29)	2506	35	0.61	0,094 (0,083 - 0,105)

**Weighted mean (weighted CI)**	4086			**0,064 (0,057 - 0,071)**

**Year 2+ on HAART**				
Senegal (24)	115	37	1.00	0,024 (-0,004 - 0,052)

**Weighted mean (weighted CI)**	115			**0,024 (-0,004 - 0,052)**

NA = Not Available

To identify evidence used as input in our analysis we carried out searches in the Cochrane Database of Systematic Reviews (inception to first quarter 2009), Ovid Medline (1996 to March 2009), EMBASE (inception to March 2008), ISI Web of Science (1992 to March 2008), conference abstracts from the International AIDS Society (2006 to 2008), the Conference on Retroviruses and Opportunistic Infections (inception to 2009) and the HIV Implementers' Meetings (2006-2008). References from relevant papers were also "hand" searched.

Search terms were; "HIV, Africa, low income countries, mortality & survival". Only English language papers were reviewed. Eligible studies were identified by the first author and their relevance confirmed by the other authors. Inclusion criteria for the structured review are: low income countries, randomised controlled trial or observational studies with minimum one year observational period, effect measures that could be converted to one year absolute risk of death, death risks stratified into baseline CD4 strata <50 or 50-199 or 200-350 cells/μl, information on number of participants in the various CD4 strata and participants older than 15 years of age.

We found no published randomised controlled trials evaluating the effect of different starting points versus placebo in low income countries. It is considered unethical to conduct such trials today. Analysis must therefore draw on the best available evidence from observational studies. We found no controlled observational studies, that is, studies comparing survival between an intervention and a control group. Effect size in terms of life expectancy can therefore only be evaluated through modelling. We identified six observational studies showing survival before initiation of HAART in low-income settings with accurate information on stratified survival according to baseline CD4 count (table 1); two from South Africa [27,28] and Thailand [29,30] and one from Uganda [31] and Gambia [32]. We found 13 observational studies showing survival after HAART in low-income settings with accurate information on stratified survival according to baseline CD4 count at start of HAART (table 2); two from Senegal [13,33], South Africa [27,34] and Botswana [35,36] and one from Cambodia [37], Côte d'Ivoire [38], Zambia [12], West-Africa [15] Haiti [39] and Malawi [14] and one multicentre study from several low income countries (ART LINC) [9]. The number of patients included in our pooled-analysis is 35 047.

The principal outcome measure in the pooled-analysis (tables 1 and 2) is one year absolute mortality risk for HIV patients with no treatment or on HAART for various baseline CD4 strata. We extracted information on absolute mortality risk from relevant studies either through assessment of estimates reported in tables or estimation from Kaplan-Meier curves. The outcome measures and 95% confidence intervals were calculated for the individual studies and the pooled-analysis examined the overall outcome (tables 1 and 2). We combined the results by calculating weighted probability (p_w_) and confidence intervals (CI) as follows:

Where p_w _is the weighted probabilities, n_i _denote number of individuals in i-th study, and p_i _represent the probability of death in the i-th study.

We combine mortality rates with a weighted mean rather than a narrative syntheses or simple mean because larger sample size (n) increases the chance the sample is representative and leads to increased precision in estimates. Hence, more weight should be given to large sample size. Weighted probabilities were used as transition probabilities in the Markov model. The effect size measure between studies in the Markov model was net life years gained and total remaining life years.

For patients not receiving HAART we assumed constant annual mortality rates of 0.796, 0.355 and 0.109 for the CD4 health states <50, 50-199 and 200-350, respectively (table 1). We applied a CD4 decline rate of 22 cells/μl, which draws on a Tanzanian observational study [40]. The one-year transition rate from one CD4 level to a lower level was calculated by dividing the baseline CD4 difference in each stratum with the annual CD4 decline rate. All treatment studies with more than one year observational period documented a peak in mortality the first year. Studies from high income countries have also documented a decreasing death risk for patients on HAART after the first year of treatment [41]. We applied a similar peak in our model, with higher HIV-related mortality rates the first year after HAART initiation, according to the findings in the pooled-analysis. From year three and onwards, we assumed constant HIV-related mortality, with year two as the annual mortality rate. We found no evidence on temporal treatment outcomes from year three and onwards to inform differently.

One-way sensitivity analyses (table 3) identified effects of applying low and high mortality rates and ages in the Markov model. High and low mortality rates in the sensitivity analyses are based on upper and lower values of the 95% weighted confidence intervals.

**Table 3:** One-way sensitivity analysis and impact on remaining life years without treatment + net benefit from HAART for HIV patients in three CD4 strata.

	Remaining life years without treatment + net benefit from HAART		
Variable	CD4 <50	CD4 50-199	CD4 200-350
Base case	0.7 + 7.2	2.0 + 7.6	4.8 + 14.5
Low HIV-related mortality rate (HAART)	0.7 + 14.5	2.0 + 9.2	4.8 + 24.3
High HIV-related mortality rate (HAART)	0.7 + 4.4	2.0 + 6.4	4.8 + 8.5
High HIV-related mortality rate (no treatment)	0.9 + 7.0	2.1 + 7.4	5.2 + 13.9
Low HIV-related mortality rate (no treatment)	0.6 + 7.4	1.9 + 7.8	4.4 + 15.1
Start age 30	0.7 + 7.5	2.0 + 8.0	4.8 + 15.9
Start age 40	0.7 + 7.0	2.0 + 7.2	4.7 + 13.2

Low and high values of mortality rates refer to upper and lower 95% CI of the combined weighted pooled estimates in table 1 and table 2.

A Tanzanian life table from 2006 with average life expectancy at age of 35 of 28.2 years for both sexes was used to adjust for the age related background mortality [24]. The life table reflects the average mortality risks of both infected and uninfected persons in various age groups, and the background mortality was therefore adjusted for the probability of death with no HAART presented in table 1. Adjusted non HIV related mortality was calculated for each age as follows: Total background mortality in the life table - (HIV related mortality * HIV prevalence).

The adult (15-50 years) HIV prevalence in Tanzania is 5.7% and it is estimated that 2.2 million people are living with HIV [25]. To our knowledge, there is only one population based study from a low income setting describing the distribution of CD4 counts in a non treated HIV population [16]. Based on this study from Malawi, we assumed that 22% and 20% out of all HIV patients have CD4 counts 200-350 and < 200 cells/μl, respectively. Since the study did not report the proportion of HIV patients with CD4 < 50 cells/μl, we assumed a lower proportion in the lowest CD4 strata due to the increased mortality rate. We assumed that among the patients with CD4 <200 cells/μl, 80% had CD4 counts 50-199 cells/μl and 20% had CD4 counts <50 cells/μl.

## Results

**Figure 2:**
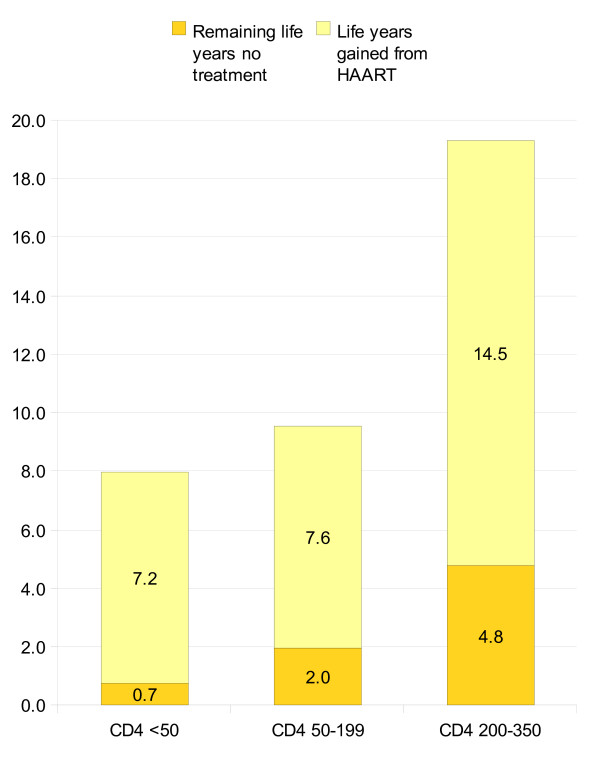
**Mean remaining life years without treatment and life years gained from HAART stratified in three baseline CD4 ranges**.

We calculated expected remaining life years without HAART and net life years gained from HAART by different CD4 starting points (figure 2). From figure 2 it can be seen that the sickest patients have 0.7 remaining life years without treatment and net life years gained from HAART is 7.2 life years. Patients with CD4 counts 50-199 cells/μl are expected to live 2.0 life years without treatment and have a net health benefit of 7.6 life years from HAART. Patients with CD4 counts 200-350 cells/μl have 4.8 remaining life years without treatment and gain an extra 14.5 life years with early treatment start.

Results from the one-way sensitivity analyses are shown in table 3, and show that outcomes are highly sensitive to assumptions about death risk for patients receiving HAART. High and low values of death risks with and without HAART were taken from the upper and lower bounds of the weighted 95% confidence intervals of the pooled-analysis. Applying the most optimistic reductions in death risks from HAART yields estimated survival of 24.3, 9.2 and 14.5 life years per person if starting HAART at CD4 200-350, CD4 50-199 and <50 cells/μl, respectively. The corresponding figures using the most pessimistic death risks were 8.5, 6.4 and 4.4 life years. The effects of death risks without treatment, and assumptions about mean age of patients were also tested. Outcomes turned out to be much less sensitive to these assumptions (table 3).

## Discussion

Our study suggests that providing HAART early is likely to yield most life years if everyone receives treatment. This is in concordance with the recommendations in the revised 2009 WHO guidelines. However, few governments in low income countries are capable of providing optimal treatment to all if everyone with CD4 < 350 cells/μl are considered to be eligible. Given the severe budget constraints low income countries face, a policy where fewer patients are considered to be eligible is the more common practice.

In terms of stratified net life years gained from HAART, we believe our study to be unique. From international published databases, we were unable to identify any studies which describe stratified net life years gained from HAART with various starting points. Five studies reported life expectancy for HIV patients when starting treatment at CD4 < 200 cells/μl and results ranged from 6.8 life years to 14.9 life years, which is comparable to our total survival (without treatment + HAART) of 9.6 life years in figure 2[17,19-22]. Four studies reported life expectancy when starting HAART at CD4 < 350 cells/μl and results ranged from 9.4 to 17.2 life years, which is slightly lower than our total survival of 19.3 life years (figure 2) [17,18,20,22]. However, our estimated health benefit from HAART was reduced to 14.5 life years (figure 2) when we adjusted for lead time bias. One study reported life years gained by increasing HAART initiation threshold from CD4 counts below 250 to 350 cells/μl, and found undiscounted survival gain to be 1.04 life years [18]. This is lower than our estimated 6.9 life years and could be explained by the fact that we apply a threshold of 200 cells/μl rather than 250 cells/μl.

The Tanzanian ART guideline from 2005, which is still being used in 2009, recommends treating asymptomatic patients when the CD4 count drops below 200 cells/μl [42]. Asymptomatic patients with higher CD4 counts and greater potential to benefit may be excluded. The revised 2009 WHO guideline considers all patients with CD4 counts below 350 cells/μl eligible for treatment, no patient groups are excluded. This goal is ambitious for resource constrained settings, and implies that up to 573 000 adult HIV patients would be eligible for HAART in Tanzania. Much more resources would be needed to scale up national ART programs to this level [43].

### Strengths and weaknesses of our analysis

A major strength of our analysis is its' simplicity and use of evidence-based transition probabilities. The disadvantage of this approach is that we were not able to model the dynamic changes in CD4 counts for patients on HAART, which has been done in several recent studies [17,19-21]. The model cannot therefore be used to look at the development of the patient cohort in more detail. For example, the analysis only includes hard endpoints (death) and not the whole spectre of clinical events and health related quality of life that affects HIV patients.

A second strength is that the analysis includes the death risk from observational studies with more than one year observational period. This enabled us to include variations in death risks during the first two years and to reduce the effect of the high mortality peak often observed during the first 6-12 months after patients have started HAART.

Death risk from year two and onwards for patients with CD4 <50 on HAART (table 2) is low and therefore the analysis yields a high benefit from HAART for this patient group. From our sensitivity analysis (table 3) we see that remaining life years varies the most for patients receiving HAART with baseline CD4 <50 or 200-350 cells/μl. This is due to few observational studies looking at absolute death risk from year two and onwards for these patients, and that the weighted confidence intervals therefore were wider.

Finally, we applied age-related background mortality in order to provide a more precise estimate of the long term effects of HAART. However, it is uncertain at what extent the pooled HIV-related mortality rates incorporate a long term epidemiological shift for HIV patients.

## Conclusion

This study demonstrates that HIV patients live longer with early start of antiretroviral treatment in low income countries, and highlights the ethical dilemma of choosing between providing HIV treatment to patients with potential best outcomes and giving fair chances to all the more severely ill patients with less potential benefit. Since low income countries have many constraints to full coverage of HAART and more people will be eligible with an early start strategy, the results of this study is a good starting point for a more transparent and reasoned debate when drawing explicit eligibility criteria during further scale up of HAART.

## Statements

### Copyright

The Corresponding Author **Kjell Arne Johansson **has the right to grant on behalf of all authors and does grant on behalf of all authors.

### Ethics approval

This study did not involve patients or sensitive information about patients and did not therefore require ethical approval.

## Competing interests

The authors declare that they have no competing interests.

## Authors' contributions

All authors fulfill the criteria of authorship and contributed collaboratively to conception and design, analysis and interpretation of data, drafting the article, revising it critically for important intellectual content and final approval of the version to be published. We assure that there is no one else who fulfils the criteria of authorship that has not been included as an author.

KAJ was principal investigator, designed the study, performed the systematic literature search, developed the Markov model, led the analysis, and was lead author for the paper. BR was a co-investigator and participated in the planning, analysis and writing of the paper. OFN sought funding for the study, contributed to design, planning, analysis and writing of the paper. All authors read and approved the final manuscript.
